# Alternative RAS in Various Hypoxic Conditions: From Myocardial Infarction to COVID-19

**DOI:** 10.3390/ijms222312800

**Published:** 2021-11-26

**Authors:** Tomas Rajtik, Peter Galis, Linda Bartosova, Ludovit Paulis, Eva Goncalvesova, Jan Klimas

**Affiliations:** 1 Department of Pharmacology and Toxicology, Faculty of Pharmacy, Comenius University, 832 32 Bratislava, Slovakia; galis13@uniba.sk (P.G.); bartosova104@uniba.sk (L.B.); jan.klimas@uniba.sk (J.K.); 2 Institute of Pathophysiology, Faculty of Medicine, Comenius University, 811 08 Bratislava, Slovakia; ludovit.paulis@gmail.com; 3 Department of Heart Failure, Clinic of Cardiology, National Institute of Cardiovascular Diseases, 831 01 Bratislava, Slovakia; eva.goncalvesova@nusch.sk

**Keywords:** hypoxia, angiotensin-converting enzyme 2, angiotensin(1-7)

## Abstract

Alternative branches of the classical renin–angiotensin–aldosterone system (RAS) represent an important cascade in which angiotensin 2 (AngII) undergoes cleavage via the action of the angiotensin-converting enzyme 2 (ACE2) with subsequent production of Ang(1-7) and other related metabolites eliciting its effects via Mas receptor activation. Generally, this branch of the RAS system is described as its non-canonical alternative arm with counterbalancing actions to the classical RAS, conveying vasodilation, anti-inflammatory, anti-remodeling and anti-proliferative effects. The implication of this branch was proposed for many different diseases, ranging from acute cardiovascular conditions, through chronic respiratory diseases to cancer, nonetheless, hypoxia is one of the most prominent common factors discussed in conjugation with the changes in the activity of alternative RAS branches. The aim of this review is to bring complex insights into the mechanisms behind the various forms of hypoxic insults on the activity of alternative RAS branches based on the different duration of stimuli and causes (acute vs. intermittent vs. chronic), localization and tissue (heart vs. vessels vs. lungs) and clinical relevance of studied phenomenon (experimental vs. clinical condition). Moreover, we provide novel insights into the future strategies utilizing the alternative RAS as a diagnostic tool as well as a promising pharmacological target in serious hypoxia-associated cardiovascular and cardiopulmonary diseases.

## 1. Introduction

Since its discovery, the alternative RAS axis made quite the entrance into the established molecular cascades and regulatory processes on various tissue and molecular levels. The range of its impact not only resides in physiological regulation and protective counter-regulation of RAS but seems to exert promising results in tissue pathologies as well, hypoxia being one of them. This review is focused on the regulatory impact that ACE2/Ang(1-7)/Mas axis exerts over cardiovascular and pulmonary systems affected by hypoxic changes. In particular, these organ systems are the relevant site of RAS activity, as they are frequently associated with the presence of local RAS. These alone are a topic of discussion as it remains elusive in which way the local systems are connected and operate together [1]. Pathological conditions that we discussed in this review are either acute, chronic or intermittent hypoxic phenotypes with limited efficiency of therapeutical strategies and serious adverse consequences on a patient’s health outcome. We also aspired to show the purpose and clinical relevance of the ACE2/Ang(1-7)/Mas axis for future pharmacological interventions. Targeting the ACE2/Ang(1-7)/Mas axis, either through the enhancement of their activity, prevention of the metabolism or administration of selective activators could reveal the great potential for novel treatment regimens that reside in the alternative arm of RAS.

## 2. The Other Side of RAS—Protective ACE2/Ang(1-7)/Mas Axis

In contrast to the classical RAS pathway which as thoroughly researched for many years [2], another branch of RAS, specifically ACE2/Ang(1-7)/Mas axis was described as an alternative non-canonical pathway. The protective arm of RAS produces effects that could be referred to as counterbalancing actions that oppose those elicited by traditional RAS AngII/AT1R signaling cascade. Some of these effects intervene with the maintenance of vascular tone [3,4], anti-inflammatory, anti-proliferatory [5] or anti-remodeling properties [6] and create a link to the protective signalization pathways.

### 2.1. Activity Mode of ACE2-Dependent Regulation

**Figure 1:**
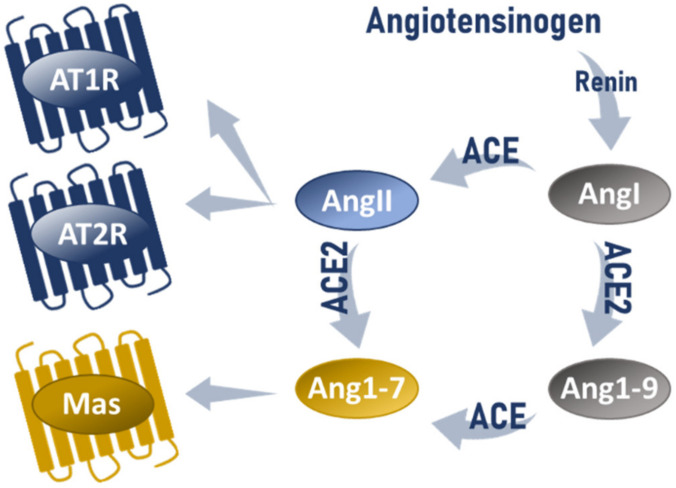
RAS cleavage cascade and the principal receptors of Ang II and Ang(1-7).

ACE2 is broadly expressed in a variety of organs and almost all kinds of tissues [7]. As a type I transmembrane protein with monocarboxypeptidase activity and 42% amino acid sequence homology in the catalytic domains with ACE [8,9,10], ACE2 manages the part of a key negative regulator of the ACE/AngII axis. The importance of ACE2 is also implemented in its function as a receptor and contact point for severe acute respiratory syndrome coronavirus (SARS-CoV) [11] and SARS-CoV-2 [12], a virus responsible for the ongoing global COVID-19 pandemic. ACE2 exerts counterregulatory actions over ACE/AngII/AT1R axis on two distinct levels: firstly, by cleavage of its main effector AngII or by limiting the availability of ACE substrate through hydrolysis of AngI and secondly, by generating the active peptide Ang(1-7). In brief, ACE2 via carboxypeptidase activity, hydrolyses octapeptide AngII, by cleavage of its C-terminal residue, resulting in the generation of heptapeptide Ang(1-7). Ang(1-7) can be also formed less efficiently via ACE-mediated conversion of Angiotensin 1-9 (Ang1-9) which was previously generated by ACE2-catalyzed hydrolysis of Angiotensin I (AngI) [8,9,13]. The key active product of the non-classical reaction cascade is Ang(1-7) whose physiological role is primarily mediated via receptor Mas (Figure 1) [14]. It is worth mentioning that another peptide product of ACE2, Ang1-9, exhibits its own physiological effects mediated through Mas receptors and AT2Rs as well. Therefore, the physiological activity of Ang1-9 does not have to end with the fact that it is an Ang(1-7) precursor but could be considered an accessory pathway of the protective RAS arm [15].

ACE2 activity is not restricted by substrate specificity for AngII but is able to cleave single-peptide residues from several bioactive peptides such as AngI, kinins (des-Arg9 bradykinin, but not bradykinin), apelins, dynorphins or neurotensins [8,16]. On the other hand, ADAM17, a disintegrin and metalloproteinase also referred to as tumor necrosis factor α (TNFα) converting enzyme, was found to mediate the proteolysis and ectodomain shedding of ACE2, thereby decreasing its activity in tissues and at the same time increasing activity of soluble ACE2 which lacks the membrane anchors as it circulates the blood [17].

### 2.2. Angiotensin (1-7) and Mas Receptor Interactions

Ang(1-7), formed directly or indirectly by ACE2 or different enzymes from the precursor peptides AngI or AngII [13,16], is an endogenous agonist for G protein-coupled receptor Mas [14]. Mas was initially identified as proto-oncogen [18] but gained a status of orphan GPCR [19] mediating Ang(1-7) signaling later on [14]. A more recent paper however documented contrasting results and suggested that Ang(1-7) does not bind to Mas directly [20], however, authors also stressed that used tissue preparations or utilization of recombinant ligand/receptor could differ from the real in vivo situation. Needless to say, there is conflicting evidence regarding the direct binding of Ang(1-7) to Mas as discussed below (for more detailed information see the review of Karnik et al., 2017 [21]). Interestingly, Mas was implicated in heteromeric interactions with other RAS receptors: forming functional interaction with AT2R [22], while inhibiting AT1R receptor response [23], which is an important fact to take into consideration whilst looking at its downstream molecular pathways. In experimental settings, the use of selective Mas antagonist A779 ((D-Ala7)-angiotensin-(1-7)) [24] as well as Mas deficiency blunted a significant amount of protective effects of the axis and promoted adverse signaling [14,25]. While these types of results do endorse the prominence of Mas in the alternative RAS axis, extended research is still indispensable. Speaking of RAS receptors, besides Mas, Ang(1-7) is known to bind AT2R [2] or the Mas-related G protein-coupled receptor member D (MRGD) [26].

### 2.3. ACE2/Ang(1-7)/Mas Axis in Cardiovascular and Pulmonary Regulation

**Figure 2:**
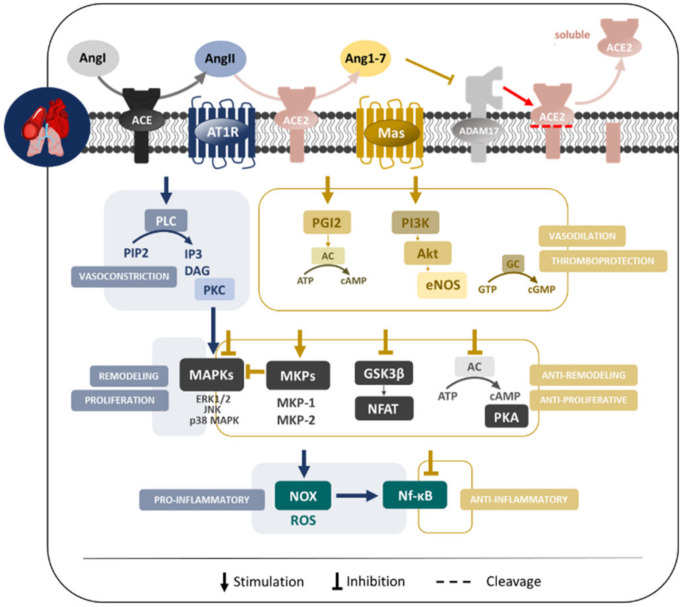
Selected molecular pathways affected by ACE2/Ang(1-7)/Mas axis in cardiovascular and pulmonary systems.

Selected molecular pathways affected by ACE2/Ang(1-7)/Mas axis in cardiovascular and pulmonary systems are graphically summarized in Figure 2.

#### 2.3.1. Heart

Local cardiac RAS activity and its interconnection with relevant molecular pathways have been a subject of interest in numerous studies that have gradually improved our understanding of their local range of actions. Specifically, on the cardiomyocyte level in which proliferative/hypertrophic stimuli are relevant factors for future cardiac remodeling, a study by Tallant et al. demonstrated Ang(1-7)-dependent inhibition of cardiomyocyte growth via mitogen-activated protein kinase (MAPK) extracellular signal-regulated kinase (ERK) 1/2 activity inhibition [27]. Ang(1-7) is generally engaged in antihypertrophic signalization, regarding the effect on the hypertrophic nuclear factor of activated T cells (NFAT) or glycogen synthase kinase 3β (GSK3β) signaling cascade [6], and involved in direct cardiac antifibrotic effects [28]. Ang(1-7) also exhibited Mas-dependent activation of endothelial NO synthase (eNOS) and Akt [29], or increased cardiac NOS expression and activity via AT2R- or bradykinin-dependent pathway [30]. Concurrently, cardioprotective actions of Ang(1-7) could be also associated with its antioxidative [31] or anti-inflammatory properties, in particular with modulation of TNF-α, interleukin-6 or interleukin-10 [32].

Notwithstanding the apparent protective effects of the alternative axis against pathological remodeling, the effects on cardiac function under physiological conditions in the settings of ACE2 gene knockout are ambiguous. In the first report by Crackower et al., ACE2-deficient hearts displayed reduced cardiac contractility, aortic and ventricular pressures, and structural changes with no signs of hypertrophy or fibrotic changes which lead them to consider the ACE2 as a potential regulator of cardiac function [33]. Conversely, Gurley et al. found no evidence of ACE2 being involved in the regulation of cardiac structure or function [34]. Other research groups were similarly not able to confirm the cardiac phenotype observed by Crackower and associates [35,36]. In line with all these findings, physiological heart function might not be ACE2-dependent, but ACE2-deficiency could rather cause higher susceptibility to cardiac injury [35,36] and favor the deleterious effects of AngII [37].

#### 2.3.2. Endothelium, Blood Vessels and Thrombosis

As the counterregulatory RAS, ACE2/Ang(1-7)/Mas axis induces vascular relaxation and improves endothelial function through the activation of eNOS and concomitantly stimulates NO/cGMP/PKG signaling pathway possibly via Akt-dependent mechanism [3,4]. As regards the coronary circulation, the data are similar in the matter that Ang(1-7) induced vasodilatation that could be blocked by Mas antagonist A-779 [38]. Anti-proliferative effect on vascular growth was proposed to be mediated by prostacyclin release, subsequent stimulation of cAMP production and suppression of AngII stimulated ERK1/2 MAPK activities as well as NAD(P)H oxidase-derived superoxide anion-mediated PI3K/Akt pathway [5,39]. Moreover, Sousa-Lopes and associates showed that Ang(1-7) negatively modulates ERK1/2 activation via stimulation of mitogen-activated protein phosphatase (MKP) 1 phosphorylation [40].

In addition to the established role in vascular tone modulation, both arms of RAS contribute to the regulation of hemostasis. While the activity of AngII on AT1R was implicated in the prothrombotic mechanisms and potentiation of platelet aggregation [41], the effects mediated through AT2R lead to thromboprotection via NO and prostacyclin production [42]. On the other hand, mounting evidence on Ang(1-7) is suggesting its potent antithrombotic effects that are mediated via the Mas [43] in both endothelial cells [3,44] and platelets [25,45]. In endothelial cells, Ang(1-7) gives rise to NO release and prostacyclin production [3,44], similarly to platelets, where Fraga-Silva et al. documented Ang(1-7)/Mas activation-stimulated NO production that was inhibited by both Mas antagonist A-779 or Mas knockout [25]. On a related note, elevated plasma levels of NO and prostacyclin were found in mice overexpressing AT2R and Mas receptors [45].

#### 2.3.3. Lungs

On the subject of local RAS activity, lungs are known to express all the major components of RAS [46] including pulmonary vasculature endothelial and smooth muscle cells, type I and II alveolar epithelial cells, bronchial epithelial cells [47,48,49,50,51,52] or bronchial smooth muscle [53]. In fact, the existence of the intrapulmonary renin–angiotensin system as a locally active system is suggested although not confirmed and therefore the possibility of lungs being a self-sufficient system in terms of expressing its own mediators without depending on their circulating precursors should be taken into consideration [46].

Although the excessive activation of classical ACE/AngII/AT1R RAS branch is responsible for many detrimental effects on the pulmonary vasculature and lung parenchyma such as vasoconstriction [4], pro-inflammatory [5,54], pro-apoptotic [55] or pro-fibrotic effects [56] as well as the acceleration of thrombosis [57], induction of oxidative stress [58] or growth-promoting actions involved in pulmonary remodeling [59], Angiotensin II-type 2 receptor (AT2R) cascade [60] likewise the alternative arm of RAS exert predominantly opposite effects. ACE2/Ang(1-7) pathway is reportedly involved in the mediation of anti-apoptotic, anti-inflammatory and anti-proliferative response via the attenuation of MAPKs activities and nuclear factor–ĸB (NF-ĸB) pathway [53,55,61,62,63]. Concurrently, the axis is suggested to regulate the activation of MAPK phosphatase-2 [64]. In particular, under ovalbumin-induced settings, Ang(1-7) exhibited anti-inflammatory properties, reduced inflammatory cell infiltration in peribronchial, perivascular or alveolar regions, inhibited phosphorylation of ERK1/2 or IĸB-α, IgE and decreased multiple pro-inflammatory cytokines and chemokines [53,63]. ACE2/Ang(1-7)/Mas was further implicated in the management of cell survival mechanisms and anti-apoptotic regulation given that ACE2/Ang(1-7) prevented alveolar epithelial cell JNK phosphorylation, caspase activation and nuclear fragmentation [61,64,65,66] or lipopolysaccharide-induced apoptosis of pulmonary microvascular endothelial cells (PMVECs) [55,62]. Furthermore, the axis exerts protective effects against lung remodeling by decreasing the collagen deposition and expression [53] and against lung fibrosis in which oxidative stress represents a relevant role along with NOX4-derived ROS-mediated RhoA/Rho kinase pathway [67,68,69]. On top of that, Ang(1-7) axis does seem to inhibit the activity of ADAM17, a shedding enzyme involved in ACE2 proteolysis and pulmonary inflammation [66].

## 3. ACE2 Deficiency-Related Pathologies Underlying Hypoxia

Hypoxia is a condition in which the body or a region of the body is deprived of an adequate oxygen supply at the tissue level [70]. During acute hypoxia, hypoxic cells temporarily shift from oxidative to anaerobic (lactate) metabolism, providing a small amount of energy [71]. Severe or prolonged hypoxia can lead to cell death [72]. In most tissues of the body, the response to hypoxia is vasodilation, thus allowing greater perfusion. By contrast, in the lungs, the response to hypoxia is vasoconstriction [73]. Ischemic hypoxia (e.g., ischemia-reperfusion injury [74]) and hypoxemic hypoxia (e.g., ARDS [75]) are well-known examples of acute hypoxic conditions. On the other hand, chronic hypoxia is caused by long-lasting reduced oxygen content in the blood [76]. Chronic hypoxia is typically observed in chronic obstructive pulmonary disease (COPD) with the development of emphysema and subsequent decreased air-exchanging capacity of lungs [77], in pulmonary hypertension (PH) accompanied by pulmonary edema [78], or heart failure (HF) characterized by the insufficient tissue perfusion and blood congestion in the pulmonary blood stream (congestive heart failure) [79].

### 3.1. Activity of ACE2-Ang(1-7)-Mas Axis in Settings of Acute Hypoxia

#### 3.1.1. Ischemia-Reperfusion Injury and Myocardial Infarction

Ischemia-reperfusion injury (I/R injury or IRI) is the tissue damage caused by the paradoxical exacerbation of cellular dysfunction and death when blood supply returns (reperfusion) to previously ischemic tissue (anoxia or hypoxia). The absence of oxygen and nutrients during the ischemic period creates a condition in which the restoration of circulation results in inflammation and oxidative damage. IRI occurs in a wide range of organs including the heart, lungs, kidney, gut, skeletal muscle and brain and may involve not only the ischemic organ itself but may also induce systemic damage to distant organs [80]. An example can be renal IRI leading to renal failure and subsequent cardiovascular pathologies (hypertension, heart failure, etc.) [81]. The renin–angiotensin system is known to be stimulated after myocardial infarction (MI) [82]. Notwithstanding the presence of Ang II is significant in MI, the role of the cardioprotective counterpart, ACE2-Ang(1-7)-Mas axis, as an early form of compensatory mechanism is unknown.

The situation surrounding ACE2/Ang(1-7) axis in the heart is slightly trickier. Renal IRI can cause degenerative changes in heart histology with the onset of oxidative stress and inflammation accompanied by increased ACE/ACE2 and Ang II/Ang(1-7) levels in heart tissue [83]. If we take a closer look at myocardial IR injury, or more precisely animal (rat and murine) models of myocardial infarction (mainly occlusions of the left anterior descending coronary artery), the results almost consistently showcase surprising findings. Three different papers [24,84,85] showed that 4 weeks after MI the protein levels of ACE2 were increased in the infarcted zone, whereas the non-infarcted zone was not affected, although neglecting Ang(1-7) levels. A study by Kassiri et al. [85], however, observed decreased mRNA ACE2 levels in the infarcted zone, which could suggest that the heart itself is in fact ACE2-deficient but the excess of ACE2 might come from the circulating blood into the damaged tissue. On the other hand, the work of Qi et al. [86], made an effort to contradict these observations, but in fact, they collected tissues from the peri-infarct region, not directly from the infarcted zone. Moreover, one experiment showed increased mRNA of ACE and ACE2 in both infarct and non-infarct regions after the same period of reperfusion [87]. Overall, all these findings are still inconclusive and need further investigation.

Despite the discrepancies in these findings, gene-modifying and treatment-utilizing animal models can shed more light on this problem. Similarly, to other I/R models, ACE2-transgenic rats showed reduced left ventricular (LV) volume, the extent of myocardial fibrosis, increased tissue levels of ACE, AngII, and collagen type I in the myocardium and increased LV ejection fraction [88]. Loss of ACE2 enhanced the susceptibility to MI, with increased mortality, infarct expansion, oxidative stress, remodeling, inflammation and adverse ventricular remodeling characterized by ventricular dilation and systolic dysfunction [85]. Treatment of ex vivo hearts by administration of 10^−8^ M Ang(1-7) into the bath solution using Langendorff technique re-established the impulse conduction during ischemia-reperfusion and reduced incidence of arrhythmias during IR, possibly due to cardiomyocyte hyperpolarization via the activation of sodium pump [89]. Activation of ACE2 by diminazene aceturate (DIZE) treatment [86,90,91,92] led to an improvement of almost all evaluated parameters (however, lacking evidence against amelioration of hypertrophy). These effects were abolished by ACE2 inhibition (“compound 16”) [84,86] or by Mas-receptor antagonist (A779) [24,88]. Nevertheless, there is still insufficient evidence regarding human studies. Just one study by Wang et al. [93] reported that patients with low serum ACE2 levels (≤1.06 ng/mL), 1 h after coronary artery bypass grafting, were associated with an increased risk of postoperative MI. To further investigate the role of ACE2-Ang(1-7)-Mas axis in clinical settings of MI, we need more human trials, especially with randomized treatments. Nonetheless, mentioned studies suggest that despite the inconsistency of observed ACE/ACE2 levels between various IR models and tissues, administration of ACE2 or any modulation of ACE2/Ang(1-7)/Mas axis has a cardioprotective potential in all of these conditions and thus such treatment could be a candidate for future human trials.

The experimental models of the lung (hind-limb constriction [94,95]) and kidney (hind-limb constriction [96] or renal pedicles [83]) IRI conducted on rats and mice lead to a rise of ACE and AngII tissue and plasma levels with a simultaneous decrease in ACE2 and Ang(1-7) levels. Only one study [97] found increased both ACE2, Mas and decreased ACE and AT1R mRNA expression in kidney tissue after 4 h of reperfusion while plasma levels of both Ang(1-7) and AngII were unchanged compared to control group. Nonetheless, the data may differ on tissue or plasma levels, experimental model (duration of reperfusion from 4 to 24 h after ischemia) or expression evaluation method (WB/ELISA/fluorescence versus qRT-PCR). Indeed, the paper by Chen et al. [94] focused on this issue and observed an immediate increase in AngII in lung tissue along with an increase in Ang(1-7) levels after 4 h of reperfusion, however, they subsequently decreased. Yet, in the plasma, AngII was increased from the beginning and Ang(1-7) was decreasing over time. Another model [83] with a longer reperfusion period (24 h) observed increased ACE/ACE2 and also Ang II/Ang(1-7) tissue ratio but unexpectedly the levels of plasma Ang(1-7) were decreased whereas the levels of plasma ACE2 did not change. To further elucidate the role of ACE2/Ang(1-7) in I/R injury in lungs and kidneys, we can make a look at rodent models with modified ACE2 expression. Both lung and kidney post-ischemic tissues exhibited inflammation (increased expression of inflammatory cytokines and enhanced leukocyte infiltration), oxidative stress and activation of apoptosis. ACE2-deficient animals showed more severe symptoms [96,98] compared to the animals with ACE2-induced overexpression eliciting only milder symptoms [95,99] in contrast to the wild-type. Similar results were obtained after the treatment with ACE2 activator DIZE in the lung [95] and renal [83] IRI or by using the AT2R agonist (“compound 21”). These results suggest that renal and lung IRI may ultimately lead to increased ACE/ACE2 tissue ratio and plasma levels, with ACE2 being an important factor in alleviating IRI symptoms.

#### 3.1.2. The Role of ACE2-Ang(1-7)-Mas in Acute Respiratory Distress Syndrome

Acute respiratory distress syndrome (ARDS) is a serious lung condition characterized by rapid onset of widespread inflammation with diffuse alveolar damage and fluid build-up in the lung alveoli [100]. The fluid keeps the lungs from filling with enough air so that a person cannot breathe, the lungs cannot move enough oxygen into the bloodstream and body tissues are restricted from sufficient perfusion [75]. ARDS is also one of the endpoints of COVID-19 disease [101]. In fact, the postulated pathomechanism of COVID-19 mentions a modulation of the ACE2-Ang(1-7) axis, however, other diseases causing ARDS can affect this axis as well (though there is no direct evidence, this idea was based on observation from the SARS epidemic in 2002 [102,103]). For instance, lethal strains of the Influenza A virus (H7N9) were observed to downregulate ACE2 and upregulate AngII levels in mice [104]. Additionally, ACE2 deficiency was found to aggravate the symptoms in this model. These findings are also supported by a human study [105] of various causes of ARDS which observed an increased Ang(1-10)/Ang(1-9) ratio, thus suggesting a decrease in ACE2 activity. On the contrary, one rat study of cigarette smoke-induced ARDS [106] detected raised levels of both ACE and ACE2, although cigarette smoke alone is able to increase ACE2 expression [107,108]. To support the idea of decreased activity of ACE2 in ARDS, the recombinant ACE2 shows activity on decreasing the occurrence of ARDS symptoms in murine [109] and rat [110] models induced by acid aspiration or lipopolysaccharide (LPS). Regarding these findings, ACE2 is expected to have beneficial effects on the prevention of ARDS, mainly based on its anti-inflammatory, as well as anti-remodeling properties.

#### 3.1.3. ACE2-Ang(1-7)-Mas Branch and COVID-19

By the fall of 2021, the COVID-19 pandemic officially reached over 220 million infected and over 4.5 million deaths worldwide. The studies showed that patients suffering from COPD [111], cardiovascular diseases [112] and diabetes [113] are more vulnerable to severe courses of COVID-19. As we previously mentioned, these pathologies are known to increase the ACE/ACE2 ratio in such patients. SARS-CoV-2 utilizes ACE2 to enter the human cells [12], however, we currently have no direct data on how the virus affects this ratio. Nevertheless, patients infected by a closely related SARS-CoV virus in 2002 showed a decrease in ACE2 expression [114,115,116], therefore, it is likely to expect that SARS-CoV-2 infection might increase the ACE/ACE2 ratio as well. It is also important to mention, that many medications might affect the expression level or activity of ACE2, even though, the gathered up-to-date data regarding the deleterious (increasing the risk of infection) or beneficial (protective effects of ACE2 on pulmonary functions) effects these might exert are conflicting. One Korean retrospective study [117] claims that the renin–angiotensin system blockers might increase the risk of infection by SARS-CoV-2, but on the other hand, the manifestation of the disease was milder and did not result in higher mortality. Nevertheless, a recently published cohort study on 8.3 million people refutes this thesis, in fact, ACEi or ARBs seems to decrease the risks of COVID-19 and overall mortality rather than the opposite [118,119]. Additionally, a difference between the ACEi and ARBs towards the ACE2/Ang(1-7) axis should be taken into consideration since ACE inhibitors deplete also ACE2 substrate resulting in a reduction of anti-inflammatory metabolites production [120].

The other important fact is that patients frequently die from suffocation by alveolar damage caused by ARDS [121,122], multi-organ failure caused by cytokine storm [122] or thromboembolism [122]. In respect of the anti-inflammatory [123] and anti-aggregant [124] properties of ACE2, current phase 2 clinical trials might bring promising results in regard to recombinant human ACE2 as a potential treatment of ARDS [125] and severe cases of COVID-19 [126]. ACE2 was found to be protective in murine [109,127,128] and rat [129] models of ARDS. Decreased ACE2 levels in lungs suffering from ARDS caused by RSV (Respiratory Syncytial Virus) were also observed in neonate children [130]. Despite insufficient data about the role of ACE2 in COVID-19 associated complications, soluble ACE2 is thought to have protective effects on a pulmonary form of the disease not only by its anti-inflammatory and anti-remodeling properties but importantly by exhibiting an anti-aggregant activity, which is important for the survival of hospitalized COVID-19 patients.

### 3.2. ACE2-Ang(1-7)-Mas Arm in Conditions of Intermittent Hypoxia

Intermittent hypoxia (also known as episodic hypoxia) is a condition in which a person or animal undergoes alternating periods of normoxia and hypoxia. Intermittent hypoxia may exert various effects on blood pressure, glucose tolerance, sympathetic activation, cognition, and inflammation depending on the dosage or experimental protocol [131,132]. Underlying mechanisms can be complicated or even unknown. One of the clinically relevant models of intermittent hypoxia is a condition known as obstructive sleep apnea (OSA). OSA is the most common sleep-related breathing disorder characterized by recurrent episodes of complete or partial obstruction of the upper airway leading to reduced or absent breathing during sleep. Relevant outcomes may include a fall in blood oxygen saturation (hypoxia), a disruption in sleep, or both [133]. It is also considered a risk factor for refractory asthma [134].

#### 3.2.1. Cardiovascular System

Chronic intermittent hypoxia (CIH) is the main pathomechanism underlying OSA which is known to affect RAS activity [134]. The utilization of rodent animal models of CIH (7 days of hypoxia followed by normoxia) to mimic the clinical onset of OSA causes many pathological changes in various organs. CIH caused an increase in ACE/ACE2 ratio in blood plasma, renal arterioles and other renal tissues, which led to an elevation of blood pressure, arterial wall thickening (arterial fibrosis), renal fibrosis and generation of reactive oxygen species (ROS) connected to oxidative stress and inflammation [135,136]. However, in a study by Lu et al., most of these effects were attenuated after administration of exogenous Ang(1-7) [137], which proposes a cardioprotective effect in OSA. Elevated levels of ACE and AngII with decreased levels of ACE2 were also observed in the hearts of mice undergoing CIH [138]. This imbalance was reversed after treatment with angiotensin receptor blocker (ARB) telmisartan. Cardiovascular benefits of ACE inhibitors and ARBs are well known in clinical practice, however, implications for the CIH are unclear. Effects of administered ACE2 or Ang(1-7) have not yet been satisfyingly examined and require further examination.

#### 3.2.2. Lungs and Pulmonary Vasculature

In the model of subchronic intermittent hypoxia/hyperoxia (after 72 h) on primary murine lung endothelial cells increased levels of ACE and reduced levels of ACE2 were observed [139]. Another in vitro model observed TGF-β-mediated epithelial-to-mesenchymal transition (EMT) in LPS-treated human bronchial epithelial cells. Angiotensin(1-7) treatment abolished the aggravation of TGF-β-mediated EMT and epithelial fibrosis upon chronic IH exposure [134]. CIH in vivo also showed a high degree of interstitial edema, alveolar atelectasis, oxidative stress, and inflammatory cell infiltration in alveolar epithelial cells [140,141] with ACE and ACE2 expression impairment. Treatment with angiotensin(1-7) was able to reverse a lung injury through attenuation of lung fibrosis [134], inflammation and oxidative stress [141].

It is generally accepted that hypoxia also exacerbates pulmonary fibrosis. Hypoxia in vitro stimulates TGF-β-mediated signaling resulting in higher collagen content, glycosaminoglycan production and accumulation of extracellular matrix in human lung fibroblasts [142,143]. This effect was also observed in chronic intermittent hypoxia in bleomycin-induced lung fibrosis in rat and murine models [144,145,146] associated with the activation of NF-κB pathway, increased levels of growth factors (TGF-β, PDGF), inflammatory cytokines (TNF, IL-1β) and overall mortality.

The effects of ACE2-Ang(1-7)-Mas axis in acute high altitude hypoxia might also be interesting, however, they have not been studied in this context. There is only a little data available and targeted exclusively on RAAS. It seems that RAAS activity is unchanged in healthy participants [147,148,149], while they are maintained in high (3000 m) altitudes. The situation changes after the further ascend to very high (above 5000 m) altitudes [150,151], where the data seem to be contradictory. It makes high altitude hypoxia a good candidate for future studies regarding both RAAS and ACE2-Ang(1-7)-Mas axis. The possible effects of the angiotensin system on high altitude pulmonary edema are also needed to be evaluated.

### 3.3. ACE2-Ang(1-7)-Mas Branch in Chronic Hypoxic Conditions

#### 3.3.1. ACE2-Ang(1-7) System Association with Heart Failure

Cardiac muscle cannot maintain essential cellular processes under hypoxemic or ischemic conditions and thus a sufficient supply of oxygen is indispensable to sustain cardiac function and viability. However, the role of oxygen in the heart is complex and goes well beyond its role in energy metabolism [152]. ACE2-Ang(1-7) system is known to be imbalanced in many cardiovascular diseases and is also affected by the hypoxic condition, as we display in this review, thus changes in the ACE/ACE2 ratio in plasma or tissue in HF patients can be expected.

The early-stage (up to 4 weeks after surgery) of rat and murine HF seems to be associated with the upregulation of both ACE/Ang II and ACE2/Ang(1-7) pathways [153,154,155,156], whereas the advanced or end-stage (more than 4 weeks after surgery) of HF tend to be associated with downregulation [153,157] of ACE2/angiotensin(1-7) and upregulation of the ACE/AngII pathway. Although these data are consistent in this time-related manner in both plasma and heart tissues, two studies, a canine congestive heart failure model [158] and a rodent HF model with ascending aortic constriction [159], do not support this theory. Our earlier study observed a similar trend as a low dose of daunorubicin, mimicking early-stage of heart failure, produced an increase in cardiac ACE2 mRNA levels whilst the higher dose markedly decreased ACE2, suggesting a direct effect of chronic daunorubicin cardiomyopathy (end-stage heart failure) on ACE2 expression [160]. ACE/ACE2 balance may determine the decompensation of HF in early stages with progressive downregulation as the disease progress.

To elucidate cardioprotective effects of Ang(1-7) in failing hearts, several rodent models of HF showed significant improvements in inflammation, oxidative stress, fibrosis, ejection fraction and overall decreased mortality in ACE2-overexpressing animals [161,162] or after treatment with rhACE2 [163] or Ang(1-7) [164]. These effects were antagonized by the blockade of the Mas receptor. ACE inhibitors are clinically acknowledged to possess such effects, however, ACE2 overexpression seems to have at least comparable cardioprotective effects similar to AT1R antagonists (irbesartan) [37] or it can be even more beneficial compared to ACE inhibitors (cilazapril) in the treatment of doxorubicin-induced cardiomyopathy [161]. The other important fact is that some AT1R antagonists (olmesartan, candesartan, losartan) also show some effects on ACE2/Ang(1-7) axis independently on AngII targeting, and some (irbesartan, valsartan) do not [165]. This idea can be supported by another finding that olmesartan seems to be superior to telmisartan in reduction of myocardial collagen deposition and adverse remodeling [156], explained by the additional effect on the ACE2/Ang(1-7) axis.

According to the clinical data, patients diagnosed with HF show an increasing trend in plasma levels of circulating soluble ACE2 in more severe NYHA groups (from NYHA-II to NYHA-IV) [159,166,167,168]. Surprisingly, LV tissue levels of ACE2 seem to decrease as the disease progresses [169] despite increased ACE2-mRNA expressions [169,170,171]; or even unchanged in late-stage patients [172]. Such phenomenon can be explained by the fact that circulating ACE2 is capable of systemic AngII cleavage, however, it lacks local (myocardial) activity. A study by Messmann et al. [170] even found decreased cardiac Mas mRNA expression. It is also important to mention that actual guidelines indicate the ACE inhibitors or ARB (among others) as first-line therapy in congestive HF patients. As we previously mentioned, these drugs are known to modulate the ACE2/ACE ratio. Unfortunately, most of the studies are completely neglecting this fact, except for one study by Epelman et al. [166] that admits this limitation and also discusses the problem of circulating versus tissue ACE2. There is also a paper by Walters et al. stating that increased ACE2 plasma activity is present in patients with atrial fibrillation since they propose that elevated plasma ACE2 activity levels are connected with increased shedding of ACE2 from the tissue into the circulation and thus increase in tissue AngII is leading to structural left atrial remodeling [173]. Therefore, plasma ACE2 was proposed as a marker of disease severity and structural remodeling in human atrial fibrillation.

The effects of failing hearts on the ACE/ACE2 ratio are as complex as in the case of COPD. Many limitations are required to be eliminated in future studies in order to evaluate the true nature of this relationship. Based on long clinical practice in the use of ACEi and ARBs, their effects on the ACE/ACE2 ratio are essential in the prognosis of HF patients. On the other hand, it makes them one of the biggest limitations of potential human trials elucidating the role of ACE2 in HF. Despite the unknown role of ACE2 in HF pathomechanism, evidence supporting its cardioprotective potential is getting stronger.

#### 3.3.2. Obstructive Coronary Artery Disease and the Role of ACE2-Ang(1-7)-Mas Axis

Coronary artery disease (CAD) or ischemic heart disease (IHD), is one of the most common cardiovascular diseases characterized by the obstruction of heart arteries with consequent reduction of blood flow into the myocardium [174]. CAD is also one of the known complications in the development of heart failure [175].

From a clinical perspective, drugs affecting RAS are commonly prescribed in CAD; however, their efficacy is in question. They are effective in the reduction of cardiovascular events compared to placebo, but not when compared with other therapeutically used drugs in CAD (calcium channel blockers, thiazide diuretics, etc.). Moreover, RAS blockers are recommended only in CAD patients with hypertension or heart failure, not in CAD patients with a lower cardiovascular risk [176,177]. Therefore, the role of RAS in CAD is not very clear, despite its impact on vascular remodeling and inflammation.

Although there is a lack of evidence demonstrating the high importance of RAS in CAD development, increased levels of circulating ACE2 were observed in patients with CAD [178,179]. On the other hand, Ang(1-7) levels remained unchanged, when compared to controls. Unfortunately, there are no interventional studies available for any impact of ACE2-Ang(1-7)-Mas axis on CAD. This link also seems to come apart after the investigation of Campbell et al. [180], where no direct connection was found between angiotensin II and angiotensin (1-7) coronary plasma levels after the treatment with various ACE inhibitors. Authors suggest that Ang(1-7) levels were increased as a result of increased metabolism of AngI, rather than by ACE2-mediated cleavage of angiotensin II.

CAD is often accompanied with the formation of atherosclerotic plaques. In experimental models of atherosclerosis (apolipoprotein E-deficient animals fed with a high-fat diet), coronary and aortic expressions of ACE2 were found to be reduced, while the levels of ACE2 mRNA were not affected [181,182]. Such conditions might be partially explained by excessive ACE2 release from vessels. In humans, ACE2 activity tends to decline with the progression of atherosclerosis [183], which is in accordance with the situation in experimental models. Knockout of ACE2 gene results in increased atherosclerotic plaque area, enhanced macrophage accumulation into the lesions, the proliferation of vascular smooth muscle cells (VSMCs), accompanied with an increase in AngII levels and increased expression of adhesive proteins, as well as increased matrix metallopeptidase 9 (MMP-9) activity [184,185]. Additionally, Mas-receptor deficiency augmented AngII-induced atherosclerosis and plaque rupture through mechanisms involving increased oxidative stress, inflammation, and apoptosis [184]. All of these complications were abolished by overexpression of ACE2 [54,186,187,188,189] and treatment with ACE2 activator (DIZE) [190,191] or subcutaneous Ang(1-7) [192]. Taken together, there is evidence regarding components of ACE2-Ang(1-7)-Mas axis involvement in CAD devolvement and progression; however, to further elucidate the therapeutic potential of modulation of this axis will require utilization of more selective tools to separate the upstream RAS effects provided by ACE inhibitors or AT1 blockers in general CAD therapy.

#### 3.3.3. Involvement of ACE2-Ang(1-7)-Mas Axis in Chronic Obstructive Pulmonary Disease (COPD)

Patients with stable COPD have an impaired ability to excrete sodium [193,194] accompanied by renal fluid retention and edema [195] enhanced by hypercapnia-induced renal vasoconstriction and antidiuresis [195]. Acute hypoxia in COPD patients does not alter or decrease renin, ACE, angiotensin or aldosterone [196,197] plasma levels. Although, renin and angiotensin II levels can be significantly elevated in the presence of an underlying condition, such as chronic hypoxia and hypercapnia [194]. Renin, angiotensin II and aldosterone levels are much higher in COPD patients with edema than in patients without edema, while sodium and water excretion is decreased significantly in edematous COPD patients. The elevation of renin, angiotensin II and aldosterone correlate with the inability to excrete sodium and water. These data suggest, that in the conjunction with hypercapnia/hypoxia-mediated disturbances in renal function, stimulation of RAAS, especially with the increase in aldosterone, may contribute to edema formation in COPD patients [198]. However, impaired ability to excrete sodium might not be simply improved by the administration of an ACE inhibitor, since ACE inhibition lowers plasma levels of aldosterone without improving sodium excretion. This suggests that the inability of patients with hypoxemic COPD to excrete sodium is not caused by their increased plasma levels of aldosterone [193].

As we mentioned in the preceding paragraphs, the ACE2-Ang(1-7)-Mas axis plays generally a protective role against the effects of angiotensin II. On the other hand, downregulation of this axis can potentiate its deleterious counterpart, the ACE-AngII-AT1 axis. Indeed, an increased ACE/ACE2 ratio is suggested to play an important role in the development of various chronic cardiovascular and pulmonary diseases. ACE2 downregulation can lead to vasoconstriction, increased platelet aggregation, inflammation, proliferation, and fibrosis [31,54,124,199]. Moreover, ACE2 downregulation was observed in arterial hypertension [31,164,200,201,202], atherosclerosis [181], coronary heart disease [203], diabetes mellitus [202,204], cardiac fibrosis/remodeling [201,205] and heart failure [31,164]. Such imbalance can also be involved in pulmonary hypertension [199,206] and chronic obstructive pulmonary disease.

Nonetheless, data on COPD and their relation to ACE2 expression are scarce and controversial. Two animal studies by Xue et al. [207] and Zhang et al. [208] suggest that ACE2 is downregulated in cigarette smoke-induced COPD, whereas characteristic inflammation and fibrosis are possible to be blocked by overexpression of ACE2 [207] or by Ang(1-7) subcutaneous infusion [208]. However, human cohort studies [209,210,211] are not so straightforward. Their findings are in contrast with animal studies, where they observed higher levels of ACE2 in patients with COPD associated with cigarette smoking compared to controls or even ex-smokers. It remains unclear whether ACE2 overexpression is an initial protective response or is associated with COPD pathophysiology, although animal studies with induced ACE2-Ang(1-7)-Mas axis activation might suggest the former. On the other hand, we cannot exclude the effects of associated comorbidities and different medications in those patients. ACE inhibitors and AT1R blockers (sartans) are known to upregulate ACE2 [212,213,214] whilst the role of inhaled corticosteroids (ICS), which are used as first-line therapy of COPD in the combination with bronchodilators, is not very clear. A study by Sinha et al. suggests that ICS upregulates ACE2 in vitro [213], while another study by Finney et al. [215] states the opposite. Human studies of Peters et al. [216] and Aliee et al. [217] also observed the decrease in ACE2 after ICS administration, although the effect of other medications cannot be refuted. There are numerous animal studies [181,218,219] suggesting statins, a group of drugs used in the treatment of dyslipidemia and atherosclerosis, might upregulate ACE2 expression [181] as well.

Moreover, it is also important to distinguish between tissue and plasma forms of ACE2. As we mentioned earlier, cleavage of membrane ACE2 into the soluble form is in part dependent on the tumor necrosis factor-α convertase ADAM17 [17], which acts as a metalloproteinase and is upregulated in various diseases accompanied with inflammation and fibrosis [220], such as HF [17,221] or interstitial lung disease [222]. ADAM17 also plays its role in SARS-CoV-2 entry into human cells [12]. Therefore, cleavage of membrane form of ACE2 may be due to the pathological upregulation of this protease, resulting in a relative decrease in local membrane ACE2 levels. Alternatively, cleavage and release of membrane ACE2 as a soluble form may provide a compensatory mechanism resulting from disease. Additionally, Ang(1–7) has a very short half-life (<9 s) and the release of soluble ACE2 from the vascular endothelium may alter systemic Ang(1–7) concentrations and the relative peripheral balance of ACE2/ACE activity [166].

Besides the studies on clinically relevant COPD models, there are also studies describing lung fibrosis and hypoxia, which are closely related to the pathophysiology of COPD [220,223,224,225]. Unfortunately, in the meantime, we can only provide data from murine [208] and rat [226] animal studies, two studies on human fetal lung fibroblasts [227,228] and one paper studying human lung fibrosis [226]. These studies suggest that lung fibrosis is accompanied by decreased ACE2 levels and subsequent treatment by exogenous ACE2 or Ang(1-7) might be beneficial. To our knowledge, no human trials connecting lung fibrosis with ACE2 have been conducted yet. The situation regarding hypoxia is similar, without any human trials. In the animal models of hypoxia-induced pulmonary arterial hypertension [229,230,231,232], treatment with recombinant ACE2 or Ang(1-7) improved symptoms of pulmonary arterial hypertension (PAH) (lowered pulmonary arterial pressure, lung fibrosis, inflammation), however, there is a lack of evidence about the involvement of ACE2 in the models including both hypoxia and COPD. It is also hard to evaluate how hypoxia alone regulates ACE2/Ang(1-7) expression. Studies on hypoxic human pulmonary artery smooth muscle cells (PASMCs) [233] and CD34^+^ cells [234] show that HIF-1-α mediates an increase in ACE2 and Mas expression, however, in the studies from Wang et al. [235] and Zhang et al. [236] on PASMCs under chronic hypoxia, the expression of HIF-1-α was increased, while ACE2 was decreased, therefore, the link between HIF-1-α and ACE2 remains elusive. Surprisingly, ACE2 activity and Ang(1-7) levels were not affected by chronic hypoxia in rats at all [237].

These results are insufficient and inconclusive in the way how COPD or lung fibrosis affects the ACE/ACE2 ratio, mainly due to different experimental methods, environmental factors or the effects of various drugs. However, targeting of ACE2/Ang(1-7)/Mas axis for pharmacological modulation as a treatment of COPD seems promising for further human trials.

#### 3.3.4. COVID-19 and the Relation with COPD

Even after an eventual decline, COVID-19 may leave some marks over the population for years to come. One of the clinical outcomes of the infection appears to be lung fibrosis [121,238,239,240,241,242,243,244,245]. Lung fibrosis is generally an irreversible condition, which can lead to PAH or COPD [224]. Indeed, fibrosis was more likely to develop in patients with a severe clinical condition, especially with high inflammatory indicators [246], however, a larger cohort needs to be engaged in clinical studies to reliably validate such results. Unfortunately, we lack accurate data on how frequently fibrosis occurs or how does it correlate with the disease severity. Since hundreds of millions of people around the globe have been infected, the possibility of increasing the rates of PAH or COPD incidence could test our medical capacities in the future.

#### 3.3.5. Role of ACE2-Ang(1-7)-Mas Arm in Pulmonary Hypertension

Pulmonary hypertension (PH) is a life-threatening condition characterized by a progressive increase in vascular resistance in the pulmonary vasculature. PH is a serious complication of several cardiovascular and pulmonary diseases, which ultimately leads to right ventricular failure and death [247,248]. The exact cause is unknown, although generally accepted pathomechanism includes a disbalance between endothelin-1 and nitrous oxide (NO) in endothelium [249] with subsequent vasoconstriction, inflammation and remodeling of lung vasculature [250]. These substances are currently the main targets of specific PH therapy [248] along with prostaglandin analogs. Current therapy is, however, only symptomatic with low or no impact on overall mortality [248]. Thus, the urge to identify novel targets for the therapy is still high.

Recently, it was shown that animal models of PAH [251,252], hypoxic pulmonary hypertension (HPH) [253] and human patients with PAH [199,206], including idiopathic pulmonary arterial hypertension (IPAH) [253], might have an increased ratio of ACE/ACE2. With the increase in pulmonary arterial pressure, ACE2 expression [254] and Ang(1-7) plasma levels [255] tend to decrease. In contrast, our research group reported an increase in lung ACE2/ACE mRNA ratio of monocrotaline-treated rats [256]. While the therapy based on blocking of the RAAS have not been proven to be significantly efficient [248] (we were able to find only one clinical study based solely on effects of ACE inhibitors on pulmonary arterial pressure [257]), aiming at the ACE2-Ang(1-7)-Mas axis seems more promising.

Several rat and murine models suggest its beneficial effects in amelioration of monocrotaline-induced PAH symptoms. Animals overexpressing ACE2 [230,251,258,259] and Ang(1-7) [229] had lower right ventricular systolic pressure and pulmonary arterial pressure, less pronounced right ventricular hypertrophy and pulmonary artery remodeling and better hemodynamics in PAH. In a swine model of HPH, similar effects were observed after treatment with recombinant ACE2 [231]. Knock-out of the ACE2 gene resulted in aggravation of the symptoms [258]. Similar results were observed in models utilizing a pharmacological modulation of ACE2 activity [260,261,262,263,264] or by injecting the exogenous Ang(1-7) [265,266]. All those beneficial effects were abolished by a blockade of Mas-receptor [251,260].

Unfortunately, there is still a lack of evidence for the clinical PH in humans. There was conducted only one pilot study using single-dose intravenous (0.2 or 0.4 mg/kg) recombinant human ACE2 as a treatment of PAH [206], although with a good clinical perspective. Nevertheless, data on clinical outcomes in PAH patients are missing.

In the following Table 1, we summarized the above-mentioned studies based on the hypoxic condition and observed changes in ACE2/Ang(1-7)/Mas axis parameters.

## 4. Clinical Perspectives and Future Directions

To this day, there is no clinically approved ACE2/Ang(1-7)-aimed pharmacotherapy, even though several experimental studies based on gene modulation and activation of the alternative RAS components or recombinant therapy demonstrated the beneficial effect of stimulating the counter-regulatory RAS, which we listed in Table 2. Although, there are some exceptions, such as ARBs or ACE inhibitors, as they often possess indirect effects on this axis (olmesartan is thought to have some direct effects) [156].

Nevertheless, there are still several ongoing and closed clinical trials evaluating the clinical benefits of intravenously administered human recombinant ACE2 (rhACE2). RhACE2 (GSK2586881) was well tolerated in IIa phase of a clinical trial in patients with ARDS [125] and PAH [267], although in the PAH study there are no published results yet. Another rhACE2 (APN01) was studied in 2nd phase trial for the treatment of COVID-19 to block viral entry and to decrease viral replication. This trial was completed in January 2021 and the results are expected soon to be published [268]. However, one trial on COVID-19 was prematurely terminated last year [126]. The idea behind the study was to bind soluble ACE2 instead of tissue ACE2 to the SARS-CoV-2 virus particle and thus decrease its virulence. Such a mechanism was seen as a promising lung- and cardioprotective approach for the administration of rhACE2.

Despite rhACE2 being possibly beneficial in the treatment of COVID-19, it could also have some drawbacks. Firstly, high costs and secondly, it has the potential of immunogenicity or interaction with S-protein-based vaccines. Two parallel clinical trials are trying to overcome some of these problems using recombinant bacterial ACE2-like enzyme (B38-CAP) [269,270]. The studies were announced in May 2020 with the possible launch of phase 1 in late 2021 or in 2022. Another option is the manufacture of the rhACE2-Fc fusion protein. The advantage of this approach in comparison to previous ones is that the fusion of sACE2 to the Fc region of human immunoglobulin can increase its avidity to viral particles, recruit immune effector functions and increase serum stability, which is a desirable quality if intended for prophylaxis [271]. One such application, SI-F019 composed of the rhACE2-Fc fusion protein, was recently assigned to phase 1 clinical trial to evaluate its safety, tolerability, and pharmacokinetic properties [272].

Immunogenicity can also be overcome by the substitution of ACE2 with Ang(1-7). Several clinical studies are preparing or recruiting participants for the 2nd phase trial for the treatment of COVID-19 [273,274,275,276]. Besides COVID-19, three other clinical trials for peripheral arterial disease [277], essential hypertension [278] and obesity-associated hypertension [279] are about to start.

However, we need to mention that all trials described in this chapter were focused on hospital treatment. For general use, especially in chronically ill patients, it is necessary to prepare orally active pharmaceuticals. Intravenous administration is easily provided in hospitals by professionals, but not in the home environment by the patients themselves. Pharmacokinetic parameters (destruction by stomach acid and intestinal enzymes, lack of absorption, short half-life) of soluble ACE2 [280] (and Ang(1-7) as well [166]) prevent its use from oral administration. There was an attempt to improve pharmacokinetic parameters of Ang(1-7) by glycosylation of this peptide [281]. Half-life and blood–brain barrier penetration was improved, although subcutaneous formulation was still required. There were also attempts at an orally active Ang(1-7). The idea was to incorporate Ang(1-7) into specialized delivery systems, including cyclodextrin-based nanoparticles in animal models of diabetes mellitus type 2 [282], hypertensive model of thrombosis [43] and erectile dysfunction in hypercholesterolemia [283]. ACE2 protein was successfully fused with non-toxic cholera toxin subunit B expressed in plant chloroplasts, which allowed ACE2 oral administration in animal models of diabetic retinopathy [284], pulmonary hypertension [285] and uveitis [286].

However, the development of small molecular drugs is only the beginning. The best pharmacological targets in ACE2-Ang(1-7)-Mas axis could be activators of ACE2 or Mas agonists. The other possible approach is an inhibition of Ang(1-7) degradation since Ang(1-7) is primarily degraded by neprilysin (or neutral endopeptidase—NEP) and aminopeptidase A (APA), secondary to ACE. Ang(1-7) is cleaved by either NEP into inactive Ang(1-4) or by ACE into another inactive molecule Ang(1-5) [287]. ACE inhibitors and NEP inhibitors are already in clinical practice, but they lack Ang(1-7) specificity, especially NEP inhibitors, such as sacubitril, target many other peptides as well. APA is a member of the hydrolase enzyme family of aminopeptidases and its main substrates are AngII and Ang(1-7). AngII is cleaved to an active peptide AngIII with similar effects to AngII. Ang(1-7) is cleaved to inactive Ang(1-4) and Ang(2-7) [287,288]. APA was found to be overactivated in MI, thus it might be a good candidate for further studies as a new pharmacological target in several cardiovascular diseases [288]. Additionally, some other compounds were studied for their inhibitory effects on APA, such as 4-amino-4-phosphonobutyric acid (4-APBA) in a mouse model of MI [288], EC33 and its dimer RB150 (firibastat) in the treatment of high blood pressure in rats [289]. There is also an effort for the treatment of lung [95] and renal [83] IRI with ACE2 activator (DIZE) and the treatment of myocardial IRI-induced necrosis by Mas agonist AVE 0991 [290]. AVE 0991 further exhibited anti-inflammatory potential in an ovalbumin-induced acute asthmatic murine model in which Mas agonist significantly reduced macrophage infiltration [291]. However, all these experiments were conducted in animal models only. To our best knowledge, no human trials using small molecular pharmacotherapy are planned yet.

## 5. Conclusions

**Figure 3:**
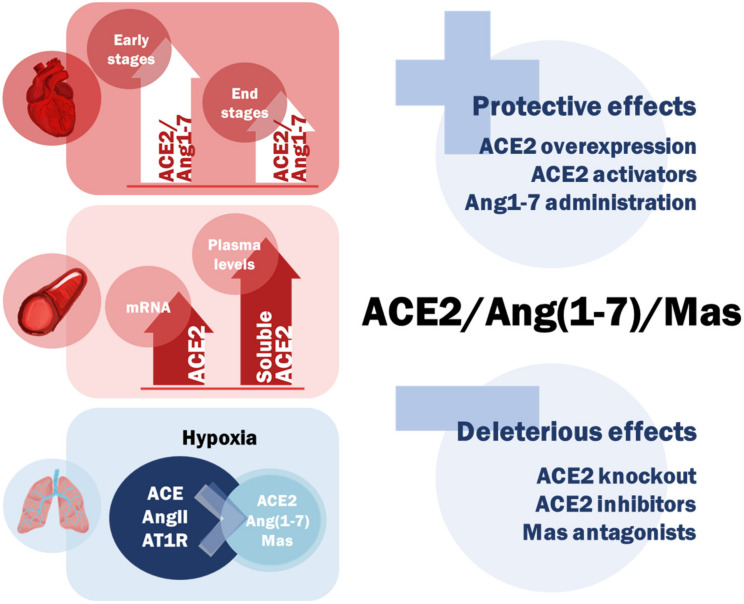
The main conclusions of the overall ACE2/Ang(1-7)/Mas axis activity in cardiovascular and pulmonary hypoxic conditions.

The main findings regarding the alternative RAS in hypoxic conditions are shown in Figure 3. In short, in both acute and chronic hypoxic conditions in CVS and the lungs, there is time-dependent regulation of the ACE2/Ang(1-7) axis, increased levels of ACE2/Ang(1-7) in the early stages in contrast to markedly reduced levels with disease progression at the end-stage. An important element of such behavior throughout the studies is the increased circulation of soluble ACE2 connected to the activity of proteolytic enzymes, particularly sheddases, such as ADAM17. However, generally, there are no major differences between most acute and chronic hypoxic conditions since the pathologies are predominantly characterized by enhanced activity of classical ACE/AngII axis and ACE2/Ang(1-7) suppression. The biggest scientific challenge lies in the fact that most of the translational and clinical studies in humans are affected by the effects of drugs that are included in the patient´s treatment regimen on the ACE2/Ang(1-7) axis or by the interaction/alteration of normal RAS activity and its components. Nonetheless, treatment potential resides in enhancing the activity of the alternative RAS arm (activators/agonists, recombinant components), which has accounted for numerous protective effects in different experimental models, contrastingly to the shutdown of this axis which promotes adverse signaling. Fluctuating plasma levels might also be an accountable prognostic/disease marker for future detection and disease outcome/treatment, particularly, increased plasma ACE2 can serve as a novel and reliable marker of acute cardiac damage. Moreover, during the time of writing this article, several clinical studies are being conducted, namely for the treatment of ARDS, COVID-19 and PAH, pointing to the fact, that the alternative RAS branch is one of the most promising neuroendocrine cascades for the future diagnosis and treatment of many hypoxia-related CVS and pulmonary diseases, including diseases such as myocardial infarction, heart failure, PAH or even COVID-19.

## Figures and Tables

**Table 1:** Overview of ACE2/Ang(1-7)/Mas parameters alteration in CVS and pulmonary hypoxia models.

Hypoxic Condition	Model	Increased Parameter	Decreased Parameter	Species	Reference
Acute hypoxia	I/R injury (after ≤4 h)	↑ pulmonary Ang(1-7) and ACE2 mRNA		mouse	[94]
		↑ renal ACE2 and Mas mRNA↓		rat	[97]
	I/R injury (after >4 h)		↓ plasma Ang(1-7), pulmonary ACE2	mouse	[94]
			↓ pulmonary Ang(1-7)	mouse	[95]
		↑ renal Ang (1-7)	↓ renal ACE2, serum Ang(1-7)	mouse	[96]
			↓ cardiac and hepatic ACE2	rat	[83]
	Myocardial infarction (after 4 weeks)	↑ cardiac (infarct zone) ACE2 and Mas		rat	[24]
		↑ cardiac (infarct zone) ACE2 activity		rat	[84]
		↑ cardiac (infarct zone) ACE2	↓ cardiac (infarct and non-infarct) ACE2 mRNA	mouse	[85]
		↑ cardiac (infarct zone) ACE2 mRNA	↓ plasma ACE2 and peri-infarct tissue ACE2 activity	rat	[86]
		↑ cardiac (infarct zone) ACE2 protein and mRNA		rat	[87]
	ARDS (SARS-CoV)		↓ ACE2	Vero E6 cells	[103]
	ARDS (H7N9 influenza A virus)		↓ pulmonary ACE2	mouse	[104]
	ARDS (various)	↑ plasma Ang(1-10)/Ang(1-9) ratio	↓ plasma ACE2, Ang(1-9), Ang (1-7)/Ang(1-9) ratio	human	[105]
	ARDS (RSV)		↓ pulmonary ACE2 expression and activity	human (age ≈ 2 years)	[130]
Intermittent hypoxia	Obstructive sleep apnoe		↓ plasma Ang(1-7)	rat	[132]
			↓ plasma Ang(1-7), renal arteriole ACE2/ACE	rat	[135]
			↓ plasma and renal Ang(1-7), renal arteriole ACE2/ACE	rat	[136]
			↓ cardiac ACE2	mouse	[138]
	Subchronic intermittent lung hypoxia		↓ ACE2 mRNA	MLECs	[139]
	Chronic intermittent lung hypoxia	↑ pulmonary ACE2 mRNA		rat	[140]
Chronic hypoxia	Heart failure (early stage)	↑ plasma and cardiac Ang(1-7), Ang(1-9), cardiac ACE2 activity		mouse	[153]
		↑ cardiac and renal ACE2		rat	[154]
		↑ plasma ACE and Ang(1-7), cardiac ACE2 mRNA		rat	[155]
		↑ plasma Ang(1-7)		rat	[156]
		↑ plasma ACE2		human	[159]
		↑ cardiac ACE2 mRNA		rat	[160]
		↑ cardiac ACE2/ACE mRNA ratio		human	[169]
	Heart failure (late stage)		↓ cardiac ACE2 activty	mouse	[153]
		↑ plasma ACE2		human	[159,167,168]
		↑ cardiac ACE2 and Mas		rat	[159]
			↓ plasma ACE2	rat	[157]
		↑ plasma ACE2 activity		dog	[158]
			↓ cardiac ACE2 mRNA	rat	[160]
		↑ cardiac ACE2 protein and mRNA	↓ cardiac ACE2/ACE mRNA ratio	human	[169]
		↑ cardiac ACE2 mRNA	↓ cardiac Mas mRNA	human	[170]
		↑ cardiac ACE2 mRNA		human	[171]
	Atherosclerosis		↓ cardiac and renal ACE2	rabbit	[181]
			↓ aortic ACE2 activity	mouse	[182]
			↓ carotid aortic ACE2 activity	human	[183]
	Coronary artery disease	↑ plasma ACE2		human	[178,179]
	Chronic obstructive pulmonary disease/Pulmonary fibrosis		↓ pulmonary ACE2 mRNA	Rat	[207]
		↑ bronchial ACE2 mRNA		human	[209]
		↑ pulmonary ACE2 mRNA		human	[210]
		↑ pulmonary ACE2		human	[211]
			↓ pulmonary ACE2 mRNA and enzyme activity	human, mouse	[226]
	Pulmonary hypertension		↓ pulmonary ACE2 mRNA	rat	[251]
			↓ pulmonary ACE2	rat	[252]
			↓ pulmonary ACE2 protein and mRNA	human, mouse	[253]
			↓ plasma ACE2	human	[254]
			↓ plasma Ang(1-7)	human	[255]
		↑ pulmonary ACE2/ACE mRNA		rat	[256]
			↓ plasma Ang(1-7), ACE2	human	[199]
			↓ plasma ACE2 activity and Ang(1-7)/Ang II ratio	human	[206]

↑ increased parameter; ↓decreased parameter.

**Table 2:** Gene modulation and experimental ACE2/Ang(1-7)/Mas pharmacological treatment in hypoxic conditions.

Hypoxic Condition	Model/ Disease	Gene Modulation Model		Pharmacological Treatment					Species	Ref.
		Downregu-lation	Upregulation	Enzyme	ACE2 Activator	Mas Agonist	ACE2 Inhibitor	Mas Ant-Agonist		
Acute hypoxia	I/R injury (after 4 h)		ACE2-Tg		DIZE				mouse	[95]
	I/R injury (after >4 h)	ACE2-KO	ACE2-Tg						mouse	[96]
		ACE2-KO							mouse	[98]
	Myocardial infarction (after 4 weeks)							A779	rat	[24]
							compound 16		rat	[84]
		ACE2-KO							mouse	[85]
					DIZE		compound 16		rat	[86]
			ACE2-Tg					A779	rat	[88]
						Ang(1-7)			rat	[89]
					DIZE				rat	[90,91,92]
	Acute respiratory distress syndrome			rACE2					mouse	[109]
				rACE2					rat	[110,129]
				rACE2					human	[125]
		ACE2 shRNA		rACE2					mouse	[127]
		ACE2-KO		rACE2					mouse	[128]
	COVID-19			rhACE2 *					human	[126,268]
Intermittent hypoxia	Obstructive sleep apnoe					Ang(1-7)			rat	[137]
	Subchronic intermittent lung hypoxia (MLECs)					Ang(1-7)			mouse	[134]
	Chronic intermittent lung hypoxia (in vivo)					Ang(1-7)			mouse	[134]
						Ang(1-7)			rat	[141]
Chronic hypoxia	Heart failure (early stage)			rhACE2				A779	mouse	[163]
	Heart failure (late stage)		ACE2-Tg						rat	[161,162]
	Atherosclerosis		ACE2-Tg						HUVECs	[54]
		ACE2-KO, Mas-KO							mouse	[184]
		ACE2 siRNA							rat	[185]
			ACE2-Tg					A779	mouse	[186]
			ACE2-Tg					A779	rabbit	[187]
			ACE2-Tg					A779	THP-1 cells	[188]
			ACE2-Tg						VSMCs	[189]
					DIZE				mouse	[190,191]
						Ang(1-7)		A779	mouse	[192]
	Chronic obstructive pulmonary disease/Pulmonary fibrosis		ACE2-Tg						rat	[207]
						Ang(1-7)			mouse	[208]
				ACE2					mouse	[226]
	Pulmonary hypertension			rhACE2					human	[206]
			Ang(1-7)-Tg						PMVECs	[229]
			ACE2-Tg						PASMCs	[230]
				rACE2					pig	[231]
			ACE2-Tg					A779	rat	[251]
		ACE2-KO	ACE2-Tg						mouse	[258]
			ACE2-Tg						mouse	[259]
					Resorcinol-naphthalein			A779	rat	[260]
					XNT				rat	[261]
					Resorcinol- naphthalein		MLN4760		rat	[262]
					Resorcinol- naphthalein		MLN4760	A779	rat	[263]
					NCP-2454				rat	[264]
						Ang(1-7)			rat	[25,265]
				rhACE2					human	[267]

* ongoing clinical trials.
